# Insights into the 3D In Vitro Permeability and In Vivo Antioxidant Protective Effects of Kiwiberry Leaf Extract: A Step Forward to Human Nutraceutical Use

**DOI:** 10.3390/ijms232214130

**Published:** 2022-11-16

**Authors:** Ana Margarida Silva, Andreia Almeida, Stefano Dall’Acqua, Francesca Loschi, Bruno Sarmento, Paulo C. Costa, Cristina Delerue-Matos, Francisca Rodrigues

**Affiliations:** 1 REQUIMTE/LAQV, Polytechnic of Porto-School of Engineering, Rua Dr. António Bernardino de Almeida, 4249-015 Porto, Portugal; 2 Department of Pharmaceutical and Pharmacological Sciences, University of Padova, Via Marzolo 5, 35121 Padova, Italy; 3 i3S, Institute for Research and Innovation in Health, University of Porto, 4200-135 Porto, Portugal; 4 INEB, Institute of Biomedical Engineering, University of Porto, 4200-135 Porto, Portugal; 5 Institute for Research and Advanced Training in Health Sciences and Technologies, CESPU, 4585-116 Gandra, Portugal; 6 REQUIMTE/UCIBIO, MedTech-Laboratory of Pharmaceutical Technology, Department of Drug Sciences, Faculty of Pharmacy, University of Porto, Rua de Jorge Viterbo Ferreira, 228, 4050-313 Porto, Portugal; 7 Associate Laboratory i4HB, Institute for Health and Bioeconomy, Faculty of Pharmacy, University of Porto, 4050-313 Porto, Portugal

**Keywords:** *Actinidia arguta* leaves, 3D intestinal model, in vivo antioxidant effects, nutraceutical ingredient

## Abstract

*Actinidia arguta* (Siebold & Zucc.) Planch. ex Miq. (kiwiberry) leaves are a source of phenolic compounds with pro-health biological effects, such as antioxidant and anti-inflammatory activities. Despite the huge number of studies reporting the composition of *A. arguta* leaves, no in vitro or in vivo studies explore its potential use as nutraceutical ingredient based on these activities. Therefore, this study aims to characterize the safety profile of kiwiberry leaf extracts using in vitro and in vivo approaches through the assessment of intestinal cell viability (Caco-2 and HT29-MTX), 3D intestinal permeation, and, most important, the redox markers, biochemical profile and liver and kidney function effects after the animal assays. Briefly, wistar rats were orally treated for 7 days with kiwiberry leaf extracts (50 and 75 mg/kg bw), water (negative control), or vitamin C (positive control). The cell viability was above 90% at 1000 μg/mL for both cells. Coumaroyl quinic acid and rutin achieved a permeation higher than 25% in the 3D intestinal model. The animal studies confirmed the extracts’ ability to increase superoxide dismutase, glutathione peroxidase, and catalase content in animals’ livers and kidneys while simultaneously decreasing the triglycerides content. This study highlighted the antioxidant capacity of kiwiberry leaf extracts, ensuring their efficacy and safety as a nutraceutical ingredient.

## 1. Introduction

*Actinidia arguta* (Siebold & Zucc.) Planch. ex Miq. (kiwiberry) is a plant with a well described beneficial impact on human health, mainly due to the different biological effects associated with its consumption, such as antioxidant, anti-inflammatory, and antidiabetic activities [1,2]. These bioactive potentials are a consequence of the high content of polyphenols that are present not only in the fruits but also in leaves [1]. In fact, according to Chinese herbal medicine, *A. arguta* is classified as a medicinal plant associated with human health benefits [3]. *A. arguta* fruit, commonly known as kiwiberry, is extremely rich in bioactive molecules, such as citric and quinic acids, gallic and chlorogenic acids, catechin, epicatechin, rutin, and quercetin, as well as vitamin C [4,5,6]. In the last years, a deep attention has been paid to kiwiberry leaves due to their richness in phenolic compounds such as neochlorogenic, chlorogenic, and cryptochlorogenic acids, protocatechuic acid, catechin, and kaempferol, which have been reported by different authors [7,8,9]. These molecules may have an in vivo pro-health impact, being an ideal candidate for the development of new nutraceutical ingredients, especially with antioxidant effects. Despite the literature available regarding the potential use of kiwiberry leaf extracts in the food and pharmaceutical fields [7,8,10,11,12,13,14], the reports of in vitro and in vivo nutraceutical effects are absent. The validation of new nutraceutical ingredients should be effectively committed with the assessment of bioactive compounds in in vivo bioaccessibility and bioavailability since these compounds may suffer biochemical reactions during ingestion, digestion, or absorption processes [15]. Therefore, bioavailability studies are fundamental steps to determine the amount of bioactive compounds that exert in vivo effects [16]. Simultaneously, it is important to take into consideration the chemical structure of the bioactive molecules and their biological interactions with other macromolecules, cells, enzymes, or microflora [17]. Recently, our research team [18] employed the response surface methodology (RSM) to determine the optimal extraction conditions of kiwiberry leaves through ultrasound-assisted extraction (UAE), achieving an optimal water extraction with a solid: liquid ratio of 10% (*w*/*v*) and an ultrasonic intensity of 30 W/m^2^ during 31.11 min [18]. The optimal extract was revealed to be rich in neochlorogenic and chlorogenic acids, caffeoylquinic acid, catechin, kaempferol-3-*O*-glucoside, and isorhamnetin-3-*O*-rutinoside [18]. However, it is now fundamental to determine the in vitro bioaccessibility and bioavailability of these compounds. In vitro studies have been increasingly used in recent years to screen potential toxic effects. The cell culture models are low cost, less laborious, and have benefits in terms of ethical concerns [19]. In particular, the in vitro intestinal models that mimic the human mucosa at the absorption level may help to interpret these dynamic and complex mechanisms [19]. These models constitute a good alternative to animal models, allowing the reproducibility of the multiple transport systems with an intermediate level of complexity and morphology conditions [20]. In recent years, 3D intestinal in vitro models have been mainly composed by a co-culture with different cells of the intestinal epithelium, namely Caco-2 and HT29-MTX [19]. Caco-2 cells simulate the human colon due to the presence of microvilli and tight junctions as well as various transporters, enzymes, and nuclear receptors [21,22], while HT29-MTX is able to simulate the goblet cells, allowing us to evaluate the mucoadhesion of carrier systems [22]. Despite all these advantages, in vivo studies are still crucial to validate the in vitro assays results, correlating the mechanisms and biological effects with the human body [15,23]. The main goal of this study was to evaluate the in vitro permeation, through a 3D intestinal model, of the kiwiberry leaf extracts and, most important, to evaluate the in vivo antioxidant effects in rats, aiming to validate a new nutraceutical ingredient. To the best of our knowledge, this is the first study that estimates the antioxidant in vivo effect of kiwiberry leaf extract.

## 2. Results and Discussion

### 2.1. In Vitro Cell Viability of Kiwiberry Leaf Extract

**Figure 1:**
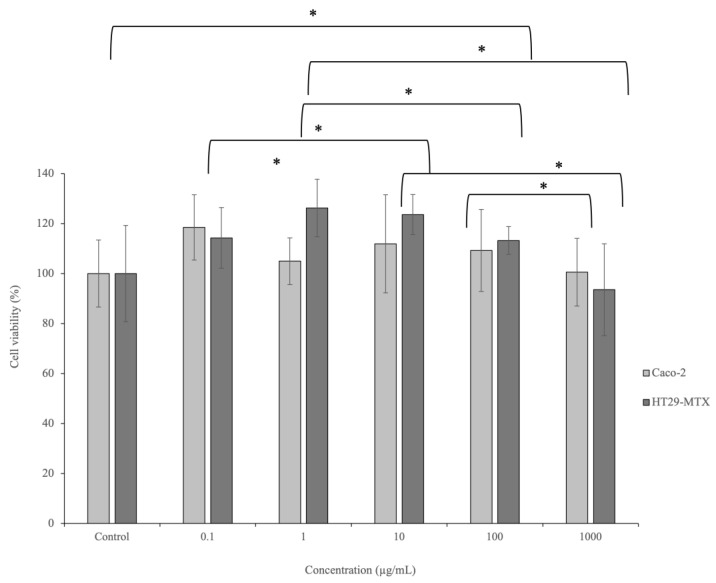
Effects of kiwiberry leaf extract exposure on Caco-2 and HT29-MTX viability at different concentrations (0.1–1000 µg/mL) as measured by the MTT assay (*n* = 3). * indicates significant differences between different concentrations of the same cell line (*p* < 0.05) according to Student’s *t*-test.

After ingestion, the phenolic compounds are subject of extensive physiological mechanisms, with the principal absorption occurring in the small intestine, although the colon plays a secondary role in the systemic absorption [24]. The results of Caco-2 and HT29-MTX viability after exposure to the kiwiberry leaf extract are represented in Figure 1.

As it is possible to observe, the extract did not affect Caco-2 cell viability, achieving results around 100%. Significant differences were observed between viabilities after exposure to the concentrations of 100 and 1000 µg/mL (*p* = 0.044). Regarding the HT29-MTX cells, the exposure to the highest tested concentration (1000 µg/mL) led to a viability of 93.54%. This viability was significantly different from the one obtained after exposure to the concentration of 10 µg/mL (*p* = 0.012). The viability after exposure to the lowest tested concentration (0.1 µg/mL) was significantly different from the viabilities achieved after exposure to 1 and 10 µg/mL (*p* < 0.024). Additionally, the viability after exposure to 1 µg/mL was significantly different from the viabilities observed after exposure to 100 and 1000 µg/mL (*p* < 0.018). These results support the absence of extract toxicity in intestinal cells.

Other authors also evaluated the toxicity of kiwiberry leaves using other extraction techniques. For examples, kiwiberry leaf aqueous extracts obtained by microwave-assisted extraction (MAE) and maceration showed similar results, with intestinal viabilities around 100% [8,10]. In the same line, Silva et al. obtained kiwiberry leaf extracts by subcritical water extraction (SWE) using different temperatures (110–160 °C) and tested their safety in intestinal cells [7]. Once again, the HT29-MTX viability was not affected, while the Caco-2 viability decreased to 80.93% after exposure to a concentration of 100 μg/mL [7].

### 2.2. 3D Intestinal Permeation Assay

**Figure 2:**
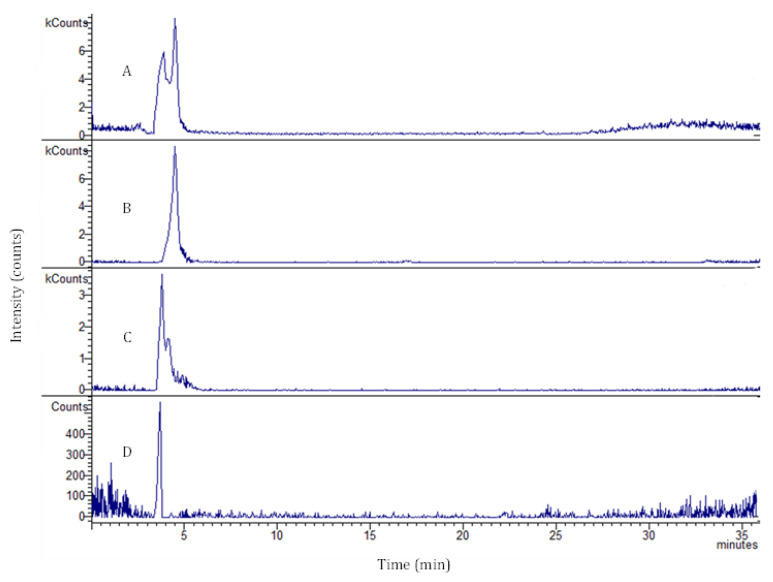
Peak ion and selective *m*/*z* traces for base peak chromatogram of the sample (trace A), chlorogenic acid (*m*/*z* 353 trace B; RT: 4.9 min), rutin (*m*/*z* 609 trace C; RT: 3.9 min), and coumaroyl quinic acid (*m*/*z* 337 trace D; RT: 3.4 min). RT: retention time.

Table 1 summarizes the different phenolic compounds identified and quantified after the 3D intestinal permeation assay at the different time points, while Figure 2 exemplifies one of the obtained chromatograms.

Coumaroyl quinic acid (RT: 3.4 min) was the principal phenolic compound identified and quantified in all time points, achieving an intestinal permeability of 34.23% at the final time point (240 min). On the other hand, rutin (RT: 3.9 min) was the principal compound determined at the first time point (6.67%), achieving a permeation of 25.40% at the final endpoint. According to Ravi et al., rutin is characterized by poor water and organic solvents solubility due to the presence of several hydroxyl groups [25]. The poor solubility of rutin affects its dissolution, absorption, and bioavailability. Furthermore, the chemical structure with multiple rings and hydroxyl groups also hinders the passage through enterocytes membranes, justifying the result obtained [25]. Chlorogenic acid (RT: 4.9 min) was the only compound detected at time points 30 min and 240 min after the 3D intestinal permeation assay, achieving a permeation of 2.49% at 240 min. This metabolite is formed by the condensation of quinic acid and *trans*-cinnamic acid [26]. The poor intestinal permeability observed may be due to possible hydrolyzation reaction that occur at intestinal cells [27]. Indeed, the compounds could be retained inside the cells, interfering in different metabolic pathways. Similar problems can be observed for flavonoids deprived of the sugar portion; neither quercetin nor kaempferol aglycones were detected, explaining the absence of permeation.

**Figure 3:**
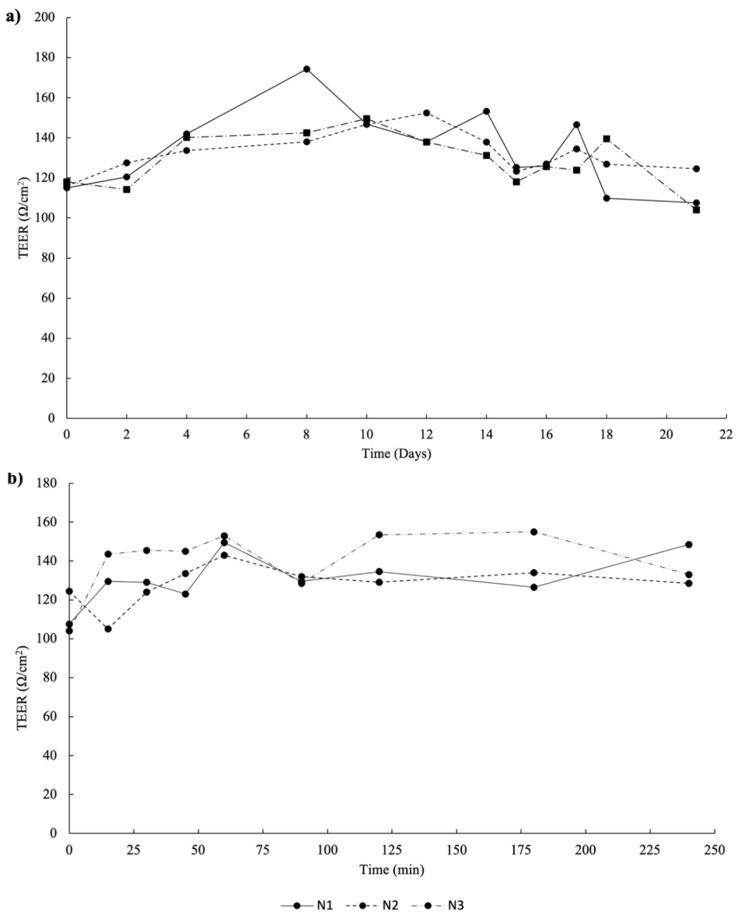
Transepithelial electrical resistance (TEER) measurements of co-culture cells (90% Caco-2 and 10% HT29-MTX) monitored during 21 days in Transwell™ membranes (**a**) and during the 240 min of the permeability assay (**b**). N1, N2, and N3 represent number of repetitions made.

The transepithelial electrical resistance (TEER) was measured for 21 days to ensure the integrity and permeability of the model. As can be observed in Figure 3a, the values increased until the 14th day, indicating cell growth. After this period, the values remained stable (120 ± 20 Ω/cm^2^), increasing at the 21st day of the experiment during the permeability assay, ranging between 104.0 Ω/cm^2^ and 148.5 Ω/cm^2^ (Figure 3b). As expected, these values are lower than other studies due to the presence of HT29-MTX cells that secret mucus, modulating the tight junctions of Caco-2 and, consequently, allowing higher inter-cellular spaces between Caco-2 and HT29-MTX [28].

### 2.3. In Vivo Animal Assays

#### 2.3.1. Effects of the Extract on the Morphological Conditions

**Figure 4:**
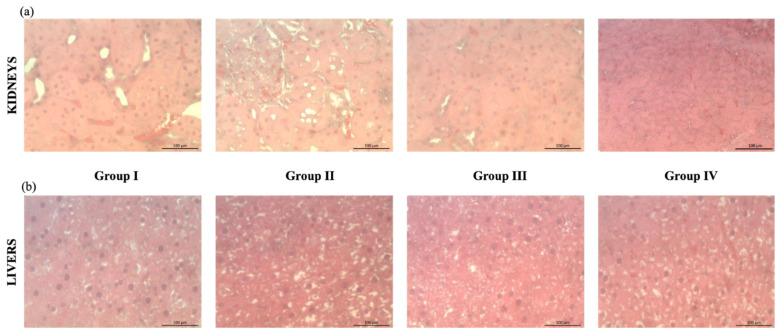
Histopathological images of kidney (**a**) and liver (**b**) tissues after hematoxylin-eosin staining: Group I—treated with water; Group II—treated with kiwiberry leaf extract 50 mg/kg bw; Group III—treated with kiwiberry leaf extract 75 mg/kg bw; Group IV—treated with vitamin C solution.

The histopathological analysis of kidney and liver sections of the different groups (Group I—treated with water; Group II—treated with kiwiberry leaf extract 50 mg/kg bw; Group III—treated with kiwiberry leaf extract 75 mg/kg bw; Group IV—treated with vitamin C solution) are represented in Figure 4. As shown in Figure 4a, the administration of kiwiberry leaf extract in different concentrations did not conduct significant alterations in the histopathological examination of kidneys. Similarly, the positive control (vitamin C) did not lead to morphological alterations. Figure 4b represents some liver sections of animals from the different groups, with the absence of morphological differences between groups being perceptible. The results obtained indicate that no alterations or inflammatory effects on the kidneys and liver were observed after animal exposure to kiwiberry leaf extracts.

#### 2.3.2. Effect of the Extract on the 2,2′-Azobis(2-amidinopropane) Dihydrochloride (AAPH)-Induced Hemolysis

Erythrocytes are blood cells highly susceptible to oxidative stress due to their content in polyunsaturated fatty acids, molecular oxygen, and ferrous ions [29]. The oxidative damage induced by hydrogen peroxide can inclusively provoke erythrocyte hemolysis [30]. The 2,2′-azobis(2-amidinopropane) dihydrochloride (AAPH) compound is able to generate free radicals by thermal decomposition in the aqueous systems, which may react with oxygen, producing peroxyl radicals that can attack the erythrocyte membrane, allowing us to study the oxidative membrane damage [29]. In this study, the influence of the kiwiberry leaf extract on the in vitro erythrocyte hemolysis induced by AAPH was evaluated. The extract provided a strong inhibition against the erythrocyte hemolysis. At the highest concentrations tested, 500 and 1000 μg/mL, the inhibition rates were 61.93% and 52.92%, respectively. In addition, the IC_50_ calculated was 251.09 μg/mL. The positive control (vitamin C) also obtained a good inhibitory activity, with values ranging between 55.31% and 74.07% after exposure to concentrations of 31.25 and 250 μg/mL, respectively.

Su et al. also studied the effect of *Pinus koraiensis* seed extract on kunming male mice erythrocyte hemolysis, reporting a inhibitory capacity in line with the present study (59%) with 0.2 mg/mL [30]. Similarly, Barreira et al. evaluated the in vitro erythrocyte hemolysis of the flowers, leaves, skins, and fruits of chestnut extracts [31], reporting an inhibitory activity between 23.3% and 95.7% (1 mg/mL) for the outer skin and fruit, respectively [31]. The results of the present study indicate that kiwiberry leaf extract has a good capacity to inhibit erythrocyte hemolysis, ensuring the protection against the oxidative damage of cell membranes.

#### 2.3.3. Effects of the Extract on Biochemical Parameters

Atherosclerosis is a consequence of hyperlipidemia, a process characterized by high levels of total cholesterol (TC) and low-density lipoprotein cholesterol (LDL-C) and associated with reduced level of high-density lipoprotein cholesterol (HDL-C) [32]. Therefore, it is imperative to search for new diet strategies to prevent and reduce metabolic disease risks. The results of triglycerides (TG), TC, HDL-C, and LDL-C serum levels of the four animal groups are summarized in Table 2.

As shown, no significant changes were observed between groups for the cholesterol data. Group II (treated with 50 mg of kiwiberry leaf extract per kg bw) had the highest TC level (88.40 mg/dL), while group IV (treated with 50 mg of vitamin C per kg bw) presented the lowest (79.81 mg/dL), without significant differences (*p* > 0.869). In the same line, group IV achieved the lowest LDL-C level, followed by group I, group II and group III, without significant differences (*p* > 0.070). Regarding HDL-C, the highest level was obtained by group I (treated with water), although no significant differences were observed between groups (*p* > 0.05).

Regarding TG levels, the values were significantly lower in the animals treated with kiwiberry leaf extracts when compared to the control groups, allowing us to conclude that the extract improved the lipidic function.

Madureira et al. studied the biochemical profile of rats after administration of solid lipid nanoparticles produced with Witepsol and Carnauba waxes and loaded with rosmarinic acid [33]. The authors reported no significant changes on TC and TG between the control group and the different solid lipid nanoparticles with Carnauba and Witepsol [33]. The TC values ranged between 43.67 and 46.67 mg/dL, while the TG values were between 107.00 and 173.30 mg/dL [33]. In another study performed by Varghese et al., the antidiabetic and antilipidemic effects of an ethanolic extract of *Clerodendrum paniculatum* flowers were evaluated in vivo [34]. The rats were divided into five groups (control, diabetic control, diabetic rats administered with glibenclamide, diabetic rats administered with 200 mg/kg extract, and normal rats treated with 200 mg/kg extract), and the authors observed that the groups treated with extract and glibenclamide had a significant increase in the HDL level and a decrease in TG, LDL, VLDL, and TG values, being in line with the control group [34]. These results are in line with the present study, highlighting the potential to prevent and reduce some diseases such as diabetes, hypertension, or hepatocarcinoma. Interestingly, the lowest concentration of kiwiberry leaf extract administrated caused a significant decrease in the serum TG, suggesting that the extract may be clinically useful for the prevention or even treatment of hyperlipidemia. Further studies are needed to confirm this effect.

#### 2.3.4. Effects of the Extract on the Antioxidant Parameters

The antioxidant activity of kiwiberry leaves has been widely reported in recent years [7,8,10,11,18], with its impact in the neutralization of oxidative free radicals being well described through decrease in the free radical scavenging enzymes, including SOD, CAT, and GSH-Px [35]. SOD is the first defense against reactive oxygen species (ROS), being present in different cells compartments [36]. CAT is also enrolled in the ROS defense, decomposing the hydrogen peroxide [37], while GSH-Px neutralizes hydroperoxides and lipid hydroperoxides [38]. Table 3 presents the effect of kiwiberry leaf extract on SOD, CAT, GSH-Px, and MDA in rat kidney, liver, and serum. The animals treated with the kiwiberry leaf extract showed a remarkable enhancement of the SOD activity in the kidneys and liver, revealing significant differences with the controls employed. The highest SOD activities in the kidneys and liver were found for group III (183.36 and 175.26 units/g protein, respectively), while group I showed the lowest results (111.01 and 82.89 units/g protein, respectively). However, in serum, group I achieved the highest SOD activity, being significantly different from the other groups (*p* < 0.011). Similarly, the serum of group I showed the best CAT activity, with significant differences with the other groups (*p* < 0.05). In the kidneys and liver, the groups treated with kiwiberry leaf extracts obtained the best CAT results, being significantly different from controls. The GSH-Px was another enzyme evaluated in the present study, and the results demonstrated that the rats administrated with 50 mg/kg bw of kiwiberry leaf extract significantly increased the GSH-Px activity (kidneys = 205.35 units/g protein, liver = 133.60 units/g protein, and serum = 64.57 units/mL protein). Regarding MDA, groups II and III exhibited the lowest levels, supporting the idea that kiwiberry leaf extracts can prevent the lipid oxidation status in the body.

Lee et al. also evaluated the effect of red onion peel and flesh on SOD, CAT, and GSH-Px in rats [39]. The animals were distributed in four groups (normal diet, normal diet with 5% red onion peel powder, normal diet with 5% red onion flesh powder, and normal diet with 5% red onion peel + flesh powder). The SOD and GSH-Px activities were measured in plasma, while the CAT activity was determined in the liver [39]. The groups administrated with 5% red onion peel powder and 5% red onion flesh powder showed the highest SOD activity; in addition, the group treated with 5% red onion flesh powder exhibited the highest GSH-Px activity [39]. Su et al. also concluded that *P. koraiensis* seed extract increased the levels of SOD and glutathione, and decreased the MDA content, in line with the present study [30]. The different concentrations of *P. koraiensis* seeds tested (250, 500, and 1000 mg/kg) led to liver SOD values between 423 and 475 units/mg protein [30]. The highest level of GSH-Px (890 units/mg protein) was reported for the normal group, while the lowest content of MDA (3.86 nmol/mg protein) was found in the group treated with 1000 mg/kg [30].

## 3. Materials and Methods

### 3.1. Chemicals

All chemicals were acquired from Sigma-Aldrich (Steinheim, Germany) and Gibco (Invitrogen Corporation, Life Technologies, UK). Caco-2 (clone type C2Bbe1) and HT29-MTX were, respectively, acquired from American Type Culture Collection (ATCC, Manassas, VA, USA) and offered from Dr. T. Lesuffleur (INSERMU178, Villejuif, France).

### 3.2. Samples

*A. arguta* leaves were collected in October 2019 in Mini-Kiwi Farm (GPS: 41.376705, -.471039). Samples were treated according to Silva et al. [18].

### 3.3. Kiwiberry Leaves Ultrasound-Assisted Extraction (UAE)

The UAE extract was obtained according to Silva et al. [18].

### 3.4. In Vitro Cell Assays

#### 3.4.1. Cell Viability Assay

The effect of the extract in Caco-2 and HT29-MTX cell lines was assessed through a 3-(4,5-dimethylthiazol-2-yl)-2,5-diphenyltetrazolium bromide (MTT) assay, according to Silva et al. [7]. Passages 70–71 and 33–34 were used, respectively, for Caco-2 and HT29-MTX. Results were presented in percentages of cell viability (% cell viability).

#### 3.4.2. 3D Intestinal Permeability Assay

The intestinal permeability was carried out in a co-culture model with Caco-2 and HT29-MTX [40]. The extract was added to the apical side of the model. A sample from the basolateral side was collected at different timepoints (0, 15, 30, 45, 60, 90, 120, 150, 180, and 240 min). Samples were analyzed by HPLC-MS assay (Section 3.5). The TEER of the model was evaluated before, during and at the end of the permeability assay using an EVOM Epithelial Volthometer Instrument equipped with a chopstick electrode (World Precision Instruments, Sarasota, FL, USA).

### 3.5. LC/DAD-ESI-MS Analysis

The phenolic compounds that permeated the intestinal 3D model were identified and quantified according to Silva et al. [41]. The samples were diluted 10 times with methanol-water (50:50) and centrifuged at 13,300× *g* rpm for 15 min. The supernatant was then transferred to a vial for the LC-MS analysis. The standard calibration curves were:

Chlorogenic acid titration curve: y = 198.01x + 20.138 (*R*^2^ = 1)
Rutin titration curve: y = 58.564x + 41.752 (*R*^2^ = 0.9998)

Regarding the multiple reaction monitoring (MRM), the values were:

Chlorogenic acid: 353.5 > 191.2
Rutin: 609.4 > 301.3
Coumaroyl quinic acid: 337.3 > 191.3

### 3.6. In Vivo Animal Study and Treatment

Young male Wistar rats of 5–6 weeks of age (140–207 g) were acquired from the Jackson Laboratory (Bar Harbor, ME, USA) and acclimatized in polypropylene cages to the environmental conditions (12 h dark/light cycle, controlled temperature (21 ± 2 °C), relative humidity (55 ± 10%) and fed ad libitum with standard pellet diet and water for 1 week before the experiment). The animal experiments were conducted according to the European Directive on Protection of Animals Used for Scientific Purposes (2010/63/EU) and ARRIVE guidelines (Animal Research: Reporting of In Vivo Experiments), following a protocol previously approved by the i3S Ethics Committee and Direção-Geral Alimentação e Veterinária (reference 2017_10) concerning the humane endpoints, appropriate husbandry, and protection of experimental animals. Groups (*n* = 6) were randomly constituted for the study experiments, receiving the following oral (gavage) treatments:

Group I–Normal control group treated with water (4 mL/kg of body weight (bw))
Group II–Kiwiberry leaf extract prepared in water (50 mg/kg bw)
Group III–Kiwiberry leaf extract prepared in water (75 mg/kg bw)
Group IV–Positive control group treated with vitamin C (50 mg/kg bw).

All groups were treated once daily for 7 days by oral gavage after a fasting period of 4 h. The body weight of each animal was recorded twice a week during the experimental period. Blood samples were collected from tail vein and a glucometer (Accu-Chek, Roche^®^ Diabetes Care, Inc., Mississauga, QC, Canada) was used to measure the glucose levels.

#### 3.6.1. Collection of Biological Samples

After 7 days, the animals were sacrificed by pentobarbital overdose (50 mg/kg bw). The organs (liver and kidneys) were removed, and the blood was collected by cardiac puncture. Serum was separated by centrifugation (20,000× *g*, 15 min, 4 °C) and stored at −80 °C for biochemical analyses [42]. The organs were dissected from each animal and subsequently mixed with 50 mM potassium phosphate, pH 7.0, and centrifuged (20,000× *g*, 15 min, 4 °C), according to the kit manufacturer’s instructions. The homogenate supernatants were analyzed by biochemical assays.

#### 3.6.2. Histopathological Studies

The tissue samples (liver and kidney) were preserved in 10% formalin. Subsequently, the tissues were dehydrated and embedded in paraffin. Then, sections of 2–3 µm thick were stained by hematoxylin-eosin. The tissue sections were observed by microscopy (IX83, Olympus Corporation, Tokyo, Japan) [43].

#### 3.6.3. Determination of In Vivo Antioxidant Parameters

The antioxidant enzyme activities, namely SOD, CAT, and GSH-Px, were evaluated in the homogenate supernatants from organs through commercial enzymatic kits from Sigma-Aldrich (Steinheim, Germany). The lipid peroxidation was determined through the determination of MDA levels using a commercial kit (Merck, Darmstadt, Germany).

#### 3.6.4. Inhibition of Erythrocyte Hemolysis in Rat Blood

The antioxidant effect of kiwiberry leaf extract was assessed through the inhibition of erythrocyte hemolysis according to the method described by Barreira et al. [31] and Barros et al. [44]. The inhibitory activity of the erythrocyte hemolysis was determined using the following formula:

Inhibition % = [(A_AAPH_ − A_S_)/A_AAPH_] × 100

where A_AAPH_ is the absorbance of the AAPH (without extract) and A_S_ is the sample absorbance.

#### 3.6.5. Biochemical Analysis

Serum was used to analyze the TC, HDL-C, and LDL-C using commercial kits from Sigma-Aldrich (Steinheim, Germany). TG levels were estimated in the blood serum through a commercial kit from Abcam (Cambridge, MA, USA).

### 3.7. Statistical Analysis

All assays were carried out in triplicate. Results were expressed as mean values and standard deviations (SD). The statistical comparisons were performed using the Student’s *t*-test and one-way analysis of variance (ANOVA), followed by the Tukey’s HSD test (with *p* < 0.05 as statistically significant), through the IBM SPSS Statistics 27.0 software (SPSS Inc., Chicago, IL, USA).

## 4. Conclusions

In the current study the safety of kiwiberry leaf extracts was demonstrated by in vitro assays, being possible to document the permeation of the principal phenolic constituents in significant amounts by a 3D intestinal model. The results of the in vivo assays elucidated the antioxidant capacity of the extracts, leading to significant increases in the liver, kidneys and serum of SOD, CAT, and GSH-Px, enzymes widely enrolled in the antioxidant defense mechanisms. Therefore, the present data provided evidence that kiwiberry leaf extract could be safely used as a nutraceutical ingredient to improve the human body antioxidant defenses and, probably, counteract the progression of other diseases, such as prediabetes. Metabolomic analysis should be performed to identify the bioactive compounds responsible for these in vivo effects.

## Figures and Tables

**Table 1:** Quantification of the kiwiberry leaf extract compounds after the 3D intestinal permeation assay. Results are expressed as mean ± SD (*n* = 3).

Time (min)	Permeability (%)		
	Coumaroyl Quinic Acid *m*/*z* = 337	Chlorogenic Acid *m*/*z* = 353	Rutin *m*/*z* = 609
15	1.04 ± 0.05	<LOD	6.67 ± 0.33
30	18.81 ± 0.94	1.79 ± 0.09	10.89 ± 0.54
45	7.99 ± 0.39	<LOD	17.01 ± 0.85
60	13.50 ± 0.68	<LOD	14.61 ± 0.73
90	19.74 ± 0.99	<LOD	14.56 ± 0.73
120	14.60 ± 0.73	<LOD	18.50 ± 0.93
180	24.32 ± 1.22	<LOD	18.30 ± 0.92
240	34.23 ± 1.71	2.49 ± 0.13	25.40 ± 1.27

LOD—Limit of detection.

**Table 2:** Biochemical results of animals treated with water (Group I), kiwiberry leaf extract (Group II—50 mg/kg bw and Group III—75 mg/kg bw), and vitamin C (Group IV). Results are expressed as mean ± SD (*n* = 3). Different letters (a,b) in the same line mean significant differences between groups (*p* < 0.05).

Biochemical Parameters	Group I	Group II	Group III	Group IV
TC (mg/dL)	82.01 ± 22.00	88.40 ± 20.40	80.66 ± 12.94	79.81 ± 15.11
HDL-C (mg/dL)	198.64 ± 46.02	191.97 ± 47.31	134.24 ± 24.83	155.09 ± 37.71
LDL-C (mg/dL)	111.37 ± 32.27	114.22 ± 30.93	131.22 ± 32.17	76.24 ± 16.52
TG (mg/dL)	304.41 ± 80.08 ^a^	70.28 ± 10.87 ^b^	86.82 ± 27.59 ^b^	229.76 ± 33.54 ^a^

**Table 3:** Antioxidant enzymes (SOD, CAT, GSH-Px, and MDA) activities in supernatants from kidney, liver, and serum of rats treated with water (Group I), kiwiberry leaf extract 50 mg/kg bw (Group II), kiwiberry leaf extract 75 mg/kg bw (Group III), and vitamin C (Group IV). Results are expressed as mean ± SD (*n* = 3). Different letters (a,b,c) in the same line mean significant differences between groups (*p* < 0.05).

Antioxidant Parameters	Group I	Group II	Group III	Group IV
**SOD**				
Kidneys (units/g protein)	111.01 ± 7.91 ^c^	168.80 ± 23.77 ^a,b^	183.36 ± 37.91 ^a^	123.13 ± 19.27 ^b,c^
Liver (units/g protein)	82.89 ± 11.36 ^b^	155.42 ± 21.61 ^a^	175.26 ± 13.99 ^a^	83.71 ± 6.06 ^b^
Serum (units/mL protein)	148.03 ± 22.92 ^a^	98.26 ± 14.49 ^b^	81.57 ± 9.63 ^b^	79.20 ± 5.76 ^b^
**CAT**				
Kidneys (nmol/min/g protein)	344,131 ± 740 ^b^	3,435,762 ± 636,996 ^a^	3,638,866 ± 685,222 ^a^	427,496 ± 78,015 ^b^
Liver (nmol/min/g protein)	4,843,676 ± 986,453 ^b,c^	7,840,180 ± 1,733,773 ^a^	7,526,357 ± 1,638,318 ^a,b^	3,762,450 ± 434,242 ^c^
Serum (nmol/min/mL protein)	73,018 ± 7656 ^a^	13,809 ± 3531 ^c^	17,369 ± 1763 ^b,c^	34,911 ± 7975 ^b^
**GSH-Px**				
Kidneys (units/g protein)	142.53 ± 16.20 ^b^	205.35 ± 46.08 ^a^	158.12 ± 15.58 ^a,b^	136.66 ± 19.62 ^b^
Liver (units/g protein)	55.21 ± 6.20 ^c^	133.60 ± 23.03 ^a^	94.36 ± 12.18 ^b^	71.34 ± 10.94 ^b,c^
Serum (units/mL protein)	36.66 ± 7.70 ^b^	64.57 ± 15.80 ^a^	26.84 ± 4.65 ^b^	20.16 ± 5.29 ^b^
**MDA**				
Kidneys (nmol/g protein)	232,452 ± 29,458 ^a^	67,598 ± 15,061 ^b^	54,566 ± 9736 ^b^	196,748 ± 31,125 ^a^
Liver (nmol/g protein)	177,919 ± 3415 ^a^	54,994 ± 5742 ^c^	44,968 ± 2907 ^c^	145,827 ± 10,063 ^b^
Serum (nmol/mL protein)	1457.93 ± 270.27 ^a^	195.44 ± 41.21 ^c^	493.13 ± 3.75 ^b,c^	746.79 ± 123.55 ^b^

## Data Availability

Not applicable.
